# Constructing a visible-light-driven photocatalytic membrane by g-C_3_N_4_ quantum dots and TiO_2_ nanotube array for enhanced water treatment

**DOI:** 10.1038/s41598-017-03347-y

**Published:** 2017-06-09

**Authors:** Qi Zhang, Xie Quan, Hua Wang, Shuo Chen, Yan Su, Zhangliang Li

**Affiliations:** 1 School of Fisheries and Life Science, Dalian Ocean University, Dalian, 116023 China; 2 Faculty of Chemical, Environmental and Biological Science and Technology, Dalian University of Technology, Dalian, 116024 China; 3 Fujian Provincial Key Laboratory of Ecology-Toxicological Effects & Control for Emerging Contaminants, Putian, 351100 China

## Abstract

Photocatalytic membranes that driven by visible light are highly desired for water treatment. Here g-C_3_N_4_ quantum dots (QDs) assembled into TiO_2_ nanotube array (TNA) membranes were fabricated for the first time as a visible-light-driven g-C_3_N_4_/TNA membrane. Benefiting from the synergistic effect of membrane filtration and photocatalysis, more than 60% of rhodamine B could be removed from water under visible light irradiation. Meanwhile, the g-C_3_N_4_/TNA membrane presented an enhanced anti-fouling ability during filtering water containing *Escherichia coli* under visible light irradiation, and a permeate flux of 2 times higher than that of filtration alone was obtained by integrated process. This study offers a promising strategy for the potential application of the visible-light-driven membranes in water treatment.

## Introduction

Membrane separation, a promising technology, has been widely applied in environmental related area due to its excellent performance, relatively low energetic cost, and nontoxicity. Membranes for water purification can offer clean water with high flexibility regarding the scale of processing^1, 2^. However, the inherent shortcoming of membrane is that the contaminants merely separated from water without further treatment. Moreover, membrane fouling caused by the deposition of compounds on the membrane surface, results in a high loss, which limits its practical applications^3, 4^. Hence, developing a multifunctional membrane is an urgent need, which can automatically overcome these drawbacks in membrane filtration for water treatment.

Most recently, photocatalytic membranes have been proposed by coupling of membrane filtration and photocatalysis. Based on the reports of Ma *et al*.^5^ and Zhang *et al*.^6^, the membranes are fabricated via the immobilization of TiO_2_ on the surface of ceramic membrane. As the contaminants in water can be degraded by photocatalysis, the integrated membrane not only presents improved removal efficiency, but also showed an enhanced fouling resistance during the filtration processes. However, most of the reported photocatalytic membranes are applied under UV light irradiation^7, 8^, which exhibit poorly photoactivity under visible light irradiation. Aiming to utilize solar energy, several attempts were made to expand the photoresponse into the visible light region^9–13^.

Graphitic carbon nitride (g-C_3_N_4_), a polymeric semiconductor with a band gap of about 2.70 eV, has been reported as an attractive metal-free photocatalyst for organic pollutant degradation, selective organic synthesis, oxygen reduction, and hydrogen production^14–16^. Recently, much interest has been focused on combining g-C_3_N_4_ with TiO_2_, which not only could extend the absorption edge into the visible light region, but could also form a heterojunction between the g-C_3_N_4_ and the TiO_2_, benefiting from the photogenerated charge carriers’ separation^17^. However, the reports are very scarce regarding the immobilization of g-C_3_N_4_ into a TiO_2_ nanotube array (TNA) membrane to fabricate a visible-light-driven g-C_3_N_4_/TNA membrane has been reported to date.

Recently, quantum dots (QDs) and their composites have been widely investigated due to their unique quantum size effects and potential applications in photoelectric devices, photocatalysis and sensors^18–21^. Moreover, the strong quantum confinement and edge effects when the QD size is down to 10 nm could render excellent optical properties. Therefore, in this work, g-C_3_N_4_ QDs were immobilized into a free-standing TNA membrane to fabricate the g-C_3_N_4_/TNA membrane and the size of g-C_3_N_4_ QDs might effectively avoid the blocking of TNA membrane. Furthermore, TNA membrane structure with straight channels and highly self-ordered arrangements could have a promising performance for water treatment. This strategy provides a very interesting opportunity for further application of visible-light-driven photocatalytic membrane in water treatment.

**Figure 1:**
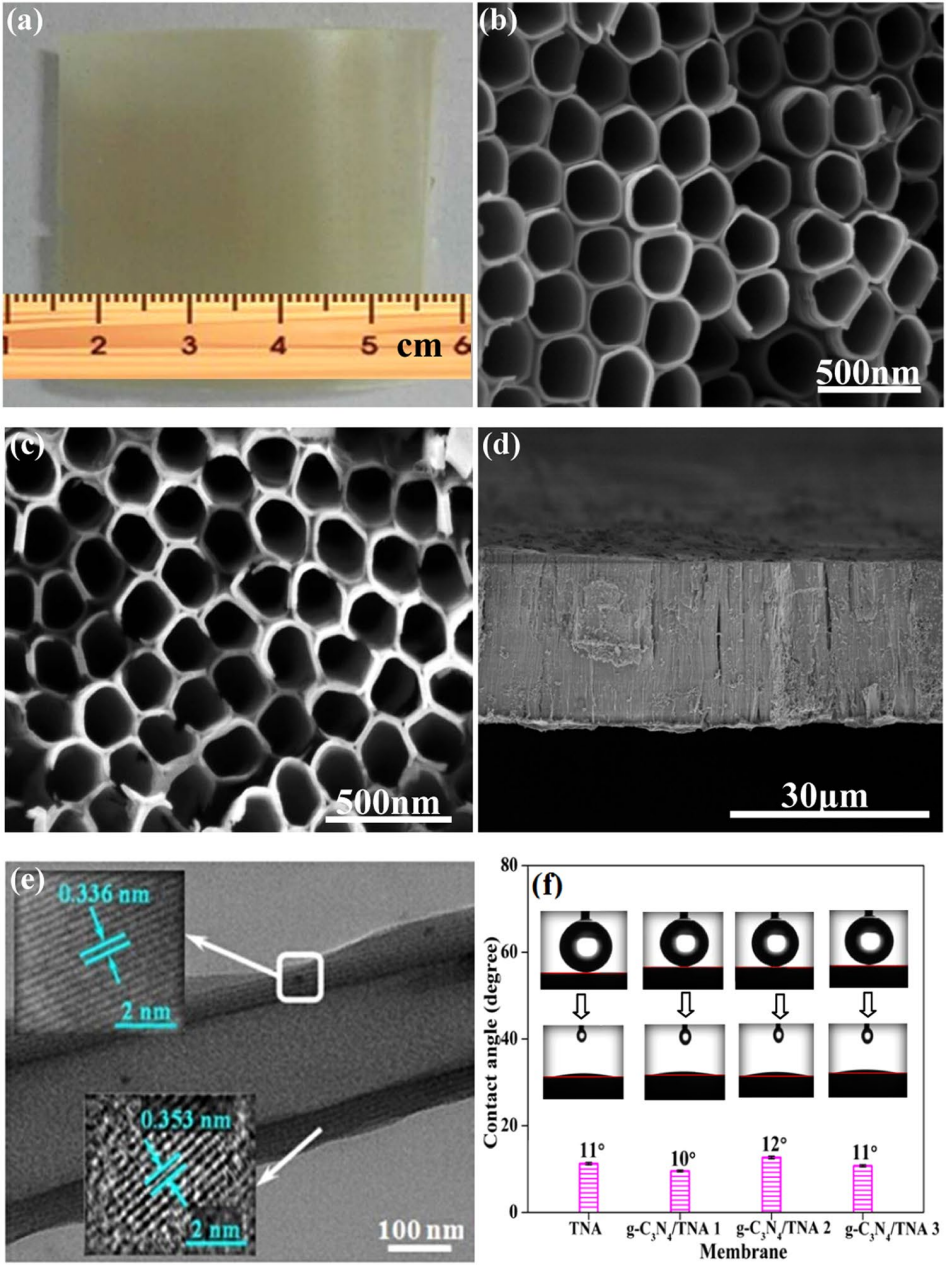
(**a**) Digital photograph of the g-C_3_N_4_/TNA membrane; SEM images of the g-C_3_N_4_/TNA membrane with (**b**) top view, (**c**) bottom view, and (**d**) cross-sectional view; (**e**) TEM images of a TiO_2_ nanotube with immobilized g-C_3_N_4_ QDs; (**f**) Water contact angle changes of TNA membrane, g-C_3_N_4_/TNA membrane 1, 2 and 3.

Figure 1a is a digital photograph of the g-C_3_N_4_/TNA membrane, showing that it is smooth and crack-free. Figure 1b–d show SEM images of the g-C_3_N_4_/TNA membrane. Figure 1b displays the top view of the g-C_3_N_4_/TNA membrane with the uniformly pores where the average inner diameter of the pores is about 190 nm and a wall thickness of 23 nm. As exhibited in Fig. 1c, the bottom side of the g-C_3_N_4_/TNA membrane is open. Fig. 1d shows the membrane consists of well-aligned nanotubes with a length of about 27 μm. Such structure of through-hole tubes could be beneficially used as a membrane. The characterizations of FT-IR, XPS and XRD can be found in Supporting Information.

Figure 1e shows a representative TEM image of the g-C_3_N_4_/TNA with a hollow tube-like structure. The high-resolution TEM image of the g-C_3_N_4_/TNA exhibited that the material was crystalline with an interplanar spacing of 0.353 nm (bottom left of Fig. 1e), which corresponded to the (101) planes of anatase TiO_2_^22–25^. The lattice spacing of 0.336 nm (top left of Fig. 1e) is indexed to the (002) planes of hexagonal g-C_3_N_4_^26, 27^. The membrane hydrophilicity of g-C_3_N_4_/TNA membrane was evaluated by measuring their water contact angles. A good hydrophilicity is beneficial to the permeating performance of membranes. Figure 1f presents that the water contact angle of all the g-C_3_N_4_/TNA membranes is lower than 12°. Meanwhile, it can be also seen that the effect of g-C_3_N_4_ QDs on g-C_3_N_4_/TNA membrane hydrophilicity is neglected.

**Figure 2:**
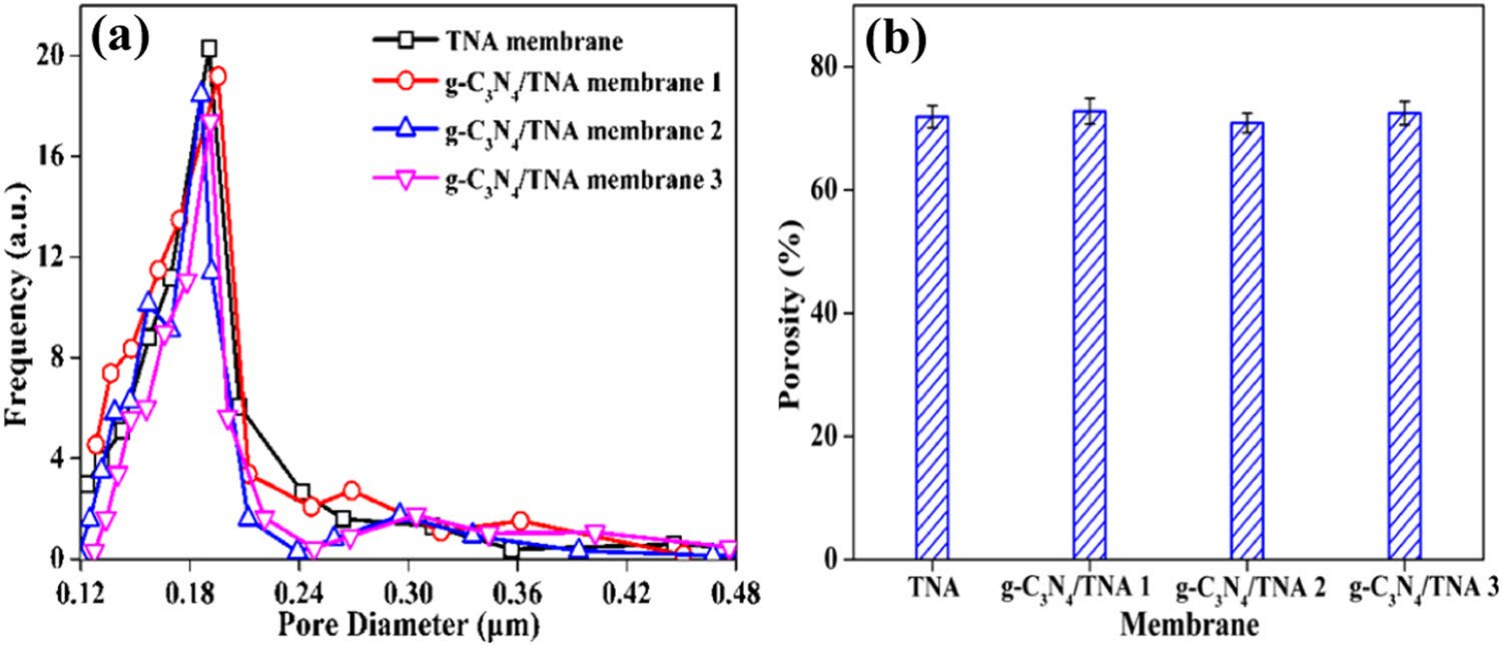
(**a**) Pore size distribution and (**b**) porosity changes of TNA membrane, g-C_3_N_4_/TNA membrane 1, 2 and 3.

Figure 2a presents the pore size distribution obtained from the g-C_3_N_4_/TNA membranes. For comparison, the pore size distribution of TNA membrane was also measured. Apparently, all the samples exhibit the same mean pore size of about 190 nm, which indicated that the deposition of g-C_3_N_4_ QDs did not markedly affect the pore size distribution of membranes and the mean pore size did not decreased with the increasing of g-C_3_N_4_ QDs deposition time. The results of mean pore size measurement were in agreement with the observation of SEM image of g-C_3_N_4_/TNA membranes. On the other hand, the porosity of TNA membrane and g-C_3_N_4_/TNA membranes is displayed in Fig. 2b. The porosity of both TNA membrane and g-C_3_N_4_/TNA membrane 1, 2 and 3 were same at about 72%. Since the porosity of traditional separation membranes is lower than 36%^28^, the high porosity of g-C_3_N_4_/TNA membrane could be attributed to its unique membrane structure including straight channels and highly self-ordered arrangements. Meanwhile, it can be seen from Fig. 2b that the effect of deposition of g-C_3_N_4_ QDs on the membrane porosity could be neglected.

**Figure 3:**
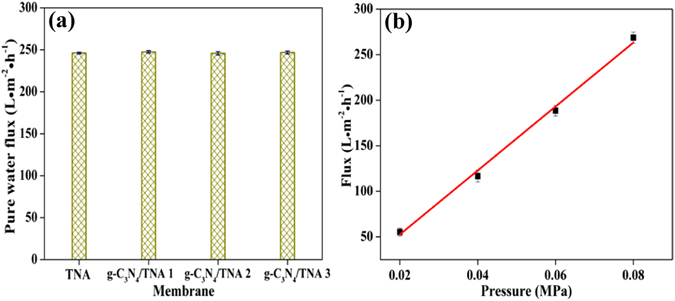
(**a**) Pure water flux (0.075 MPa) of TNA membrane, g-C_3_N_4_/TNA membrane 1, 2 and 3; (**b**) pure water flux of g-C_3_N_4_/TNA membrane 3 as a function of transmembrane pressure.

The membrane permeability was evaluated by testing pure water flux of membranes. As shown in Fig. 3a, compared with the TNA membrane, the pure water flux of all the g-C_3_N_4_/TNA membranes is about 250 L · m^−2^ · h^−1^ (0.075 MPa). This indicated that the effect of g-C_3_N_4_ QDs immobilization on the pure water flux of TNA membrane could be negligible under present experimental conditions. Figure 3b exhibits the pressure dependence of pure water flux for g-C_3_N_4_/TNA membrane. The flux increased linearly with applied pressures (0.02~0.08 MPa), indicating a high compressive resistance of the membranes used for filtration.

**Figure 4:**
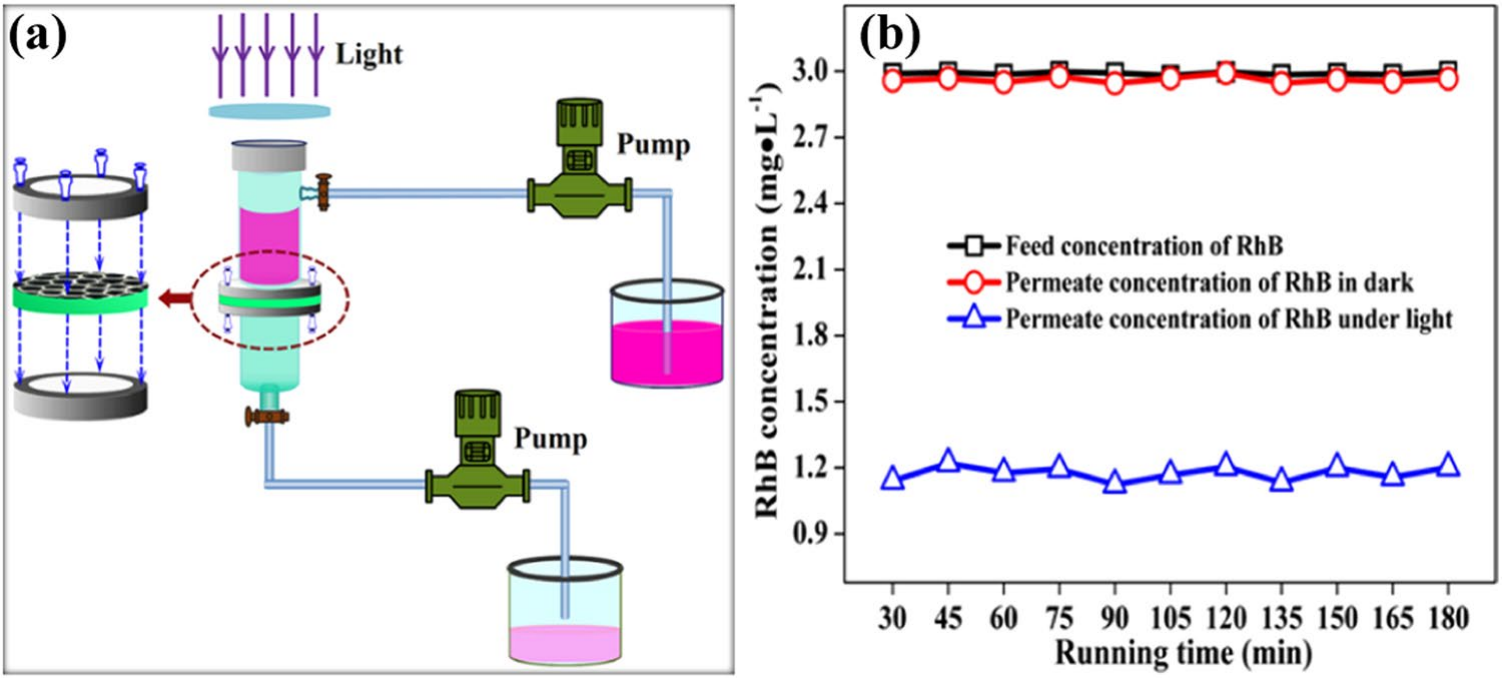
(**a**) Schematic representation of the set-up for membrane performance tests; (**b**) The performance of g-C_3_N_4_/TNA membrane 3 for RhB removal.

Figure 4a presents the experimental set-up of RhB removal using the g-C_3_N_4_/TNA membrane 3 under visible light irradiation. The performance of g-C_3_N_4_/TNA membrane 3 for RhB removal is shown in Fig. 4b. In dark, the permeate concentration of RhB was the same as the feed concentration, indicating that the membrane separation alone could not remove RhB. However, under visible light irradiation, more than 60% of RhB was removed. This indicated that the g-C_3_N_4_/TNA membrane 3 exhibited an attractive synergistic effect of membrane filtration and photocatalysis for RhB removal under visible light irradiation. In previous reports, Albuet al.^29^ demonstrated that TiO_2_ nanotube membrane can be used as a flow-through photoactive membrane for pollutant removal under UV light illumination, Liao *et al*.^30^ reported that a dramatic increase in photocatalytic activity is obtained when CdS nanoparticles are combined with TiO_2_ nanotube membrane under simulated sunlight irradiation.

In order to further investigate the mechanism of RhB photodegradation under visible light irradiation, radical trapping experiments were performed using 10 mM p-benzoquinone (BQ, ·O_2_^−^ scavengers), triethanolamine (TEOA, holes scavengers) and tert-butyl alcohol (TBA, ·OH scavengers). Compared to the RhB photocatalytic process without scavengers, the presence of TEOA and TBA scarcely affected the photocatalytic activity of the g-C_3_N_4_/TNA membrane, whereas the photocatalytic activity was greatly suppressed by the addition of BQ. This result indicated that ·O_2_^−^ is the most active species in RhB photodegradation, where holes and ·OH play a less crucial role.

**Figure 5:**
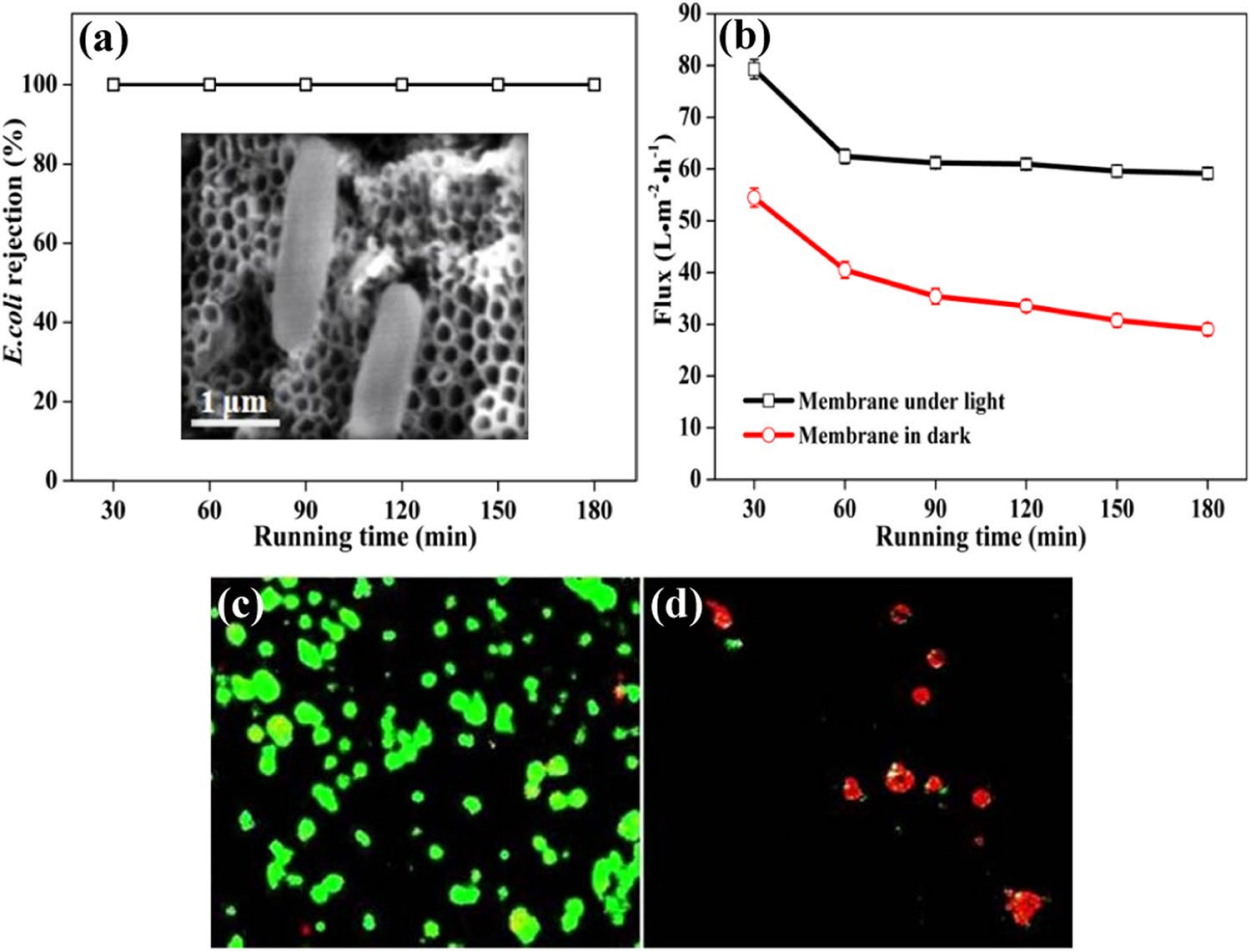
(**a**) The rejection of *E. coli* by g-C_3_N_4_/TNA membrane 3; (**b**) *E. coli* flux in outlet of g-C_3_N_4_/TNA membrane 3 with and without visible light irradiation; The fluorescent microscopic images of *E. coli* on the membrane surface (**c**) without and (**d**) with light irradiation.

To evaluate the antifouling performance of the g-C_3_N_4_/TNA membrane, the removal of *E. coli* was also carried out. Meanwhile, the permeation flux of *E. coli* was measured with and without visible light irradiation. As shown in Fig. 5a, since the size of *E. coli* was larger than that of mean pore size of the g-C_3_N_4_/TNA membrane, all the *E. coli* could be removed in outlet by membrane filtration. As presented in Fig. 5b, compared with the permeation flux continually declined to 30 L · m^−2^ · h^−1^ after 180 min in dark, the permeation flux of *E. coli* was stable at about 60 L · m^−2^ · h^−1^ from 60 to 180 min under visible light irradiation. This enhancement of the permeation flux could be ascribed to the synergistic effect of membrane filtration and photocatalysis.

The antibacterial effect on the g-C_3_N_4_/TNA membrane surface without and with visible light irradiation was further confirmed by the fluorescence microscopy images as shown in Fig. 5(c) and (d). The viable cells appear green color under a fluorescent microscope on the surface of g-C_3_N_4_/TNA membrane in the dark (Fig. 5c). However, as shown in Fig. 5d, under light irradiation, the same membrane exhibited the capability of killing *E. coli* as manifested in the higher portion of dead cells (red color) on its surface. This demonstrated that *E. coli* formed “biofouling layer” by massively gathering on the surface of g-C_3_N_4_/TNA membrane through the membrane filtration alone. On the contrary, the number of *E. coli* obviously decreased under visible light irradiation, which benefited for reducing the membrane fouling^31^. Based on the results described above, the reason of the enhanced performance of g-C_3_N_4_/TNA membrane for water treatment is that such an electron transition between g-C_3_N_4_ and TiO_2_ can reduce the recombination of charge carriers and prolong the charge lifetime^31–35^.

In summary, a facile approach has been proposed to fabricate a novel visible-light-driven photocatalytic membrane by immobilizing g-C_3_N_4_ QDs in a highly ordered TNA membrane. Benefiting from an attractive heterostructure between g-C_3_N_4_ and TiO_2_ leading to a unique photogenerated charge separation, meanwhile, since the g-C_3_N_4_/TNA membrane has a distinctive well-ordered nanotube structure with a mean pore size of 190 nm, this membrane had an impressive performance for *E. coli* removal and enhanced anti-fouling capability. Especially, the distinguish property of this obtained g-C_3_N_4_/TNA membrane is that it could be used under visible light irradiation. This visible-light-driven photocatalytic membrane shows extensive and attractive ability for water treatment.

## Methods

### Preparation of g-C_3_N_4_ QDs

Grinded bulk g-C_3_N_4_ powders were heated at 500 °C for 2 h to fabricate g-C_3_N_4_ nanosheets. Then, the g-C_3_N_4_ nanosheets (0.05 g) were treated in concentrated H_2_SO_4_ (10 mL) and HNO_3_ (30 mL) for 16 h with ultrasonication. Afterwards, the obtained g-C_3_N_4_ nanoribbons were washed with deionized water (200 mL) to remove the acids. Finally, the g-C_3_N_4_ nanoribbons were carefully redispersed in 16 mL deionized water under ultrasonication. The suspension was transferred to a Teflon-lined autoclave and heated at 200 °C for 10 h. After cooling to room temperature, the final product of yellowish g-C_3_N_4_ QD solution (about 0.14 g/L) was obtained^36^.

### Preparation of g-C_3_N_4_/TNA membranes

A vertically oriented and free-standing g-C_3_N_4_/TNA membrane was fabricated via potentiostatic anodization in a two-electrode electrochemical cell. The anodization was performed by using a commercial Ti foil (purity 99.7%) as a working electrode, and a Pt plate as a counter electrode in an electrolyte composed of 0.5 wt.% NH_4_F and 2 vol.% deionized water in ethylene glycol at 60 V for 4 h. Then, the electrodeposition of g-C_3_N_4_ QDs was performed using an electrophoresis apparatus (DYY-6C) where the pre-anodized Ti foil and Pt plate were connected with the negative pole and positive pole of the electrophoresis apparatus, respectively. The electrolyte solution consisted of isopropyl alcohol (100 mL), magnesium nitrate (5 mg), and g-C_3_N_4_ QDs (0.14 g/L, 10 mL). Electrodeposition at 20 V was maintained for a duration of 1 h (g-C_3_N_4_/TNA membrane 1), 2 h (g-C_3_N_4_/TNA membrane 2) or 3 h (g-C_3_N_4_/TNA membrane 3), respectively. Subsequently, the g-C_3_N_4_/TNA substrate was anodized for the second time at 60 V and at room temperature for 4 h. Finally, a larger anodic voltage (150 V) was applied for 5 min to get a through-hole g-C_3_N_4_/TNA membrane. The obtained samples were annealed at 500 °C for 2 h.

### Characterization

The morphology of the g-C_3_N_4_/TNA membrane was analyzed using scanning electron microscopy (SEM, Quanta 200 FEG) and transmission electron microscopy (TEM, JEM-2100F). The crystallinity of the sample was determined by X-ray diffraction (XRD) using a diffractometer with Cu Kα radiation (Shimadzu LabX XRD-6000). BET surface area and pore volume distribution were analyzed using an automated surface area and pore size analyzer (QuantachromeAutosorb-1 MP). The UV-vis absorption spectrum of the g-C_3_N_4_/TNA membrane was investigated using UV-vis diffuse reflectance spectroscopy (DRS) (Shimadzu UV-2450). Fourier transform infrared spectra (FTIR) of the samples were obtained in KBr pellets on a Nicolet 5DXC IR spectrometer (Nicolet, Madison). X-ray photoelectron spectroscopy (XPS, ESCALAB250) was used to analyze the elemental composition of the g-C_3_N_4_/TNA membrane. The photoluminescence spectrum of the sample was measured at room temperature using a 380 nm excitation wavelength (F-4500). Membrane pore size distribution was measured by using a Porometer (Porolux 1000) under a room temperature of 25 °C. The water contact angle of the g-C_3_N_4_/TNA membranes was tested using a contact angle and surface tension measurement system (Physics Instruments Ltd., Germany).

### Measurement of pore size and pure water flux

The permeate flux of membranes was carried out using the ultrapure water (18.2 Ω · cm) under different transmembrane pressures. The pure water flux of g-C_3_N_4_/TNA membrane is calculated with the following formula:

1 $$J=\,\frac{V}{Stp}\,$$

where *J* is the pure water flux, *V* is the water volume permeating membrane in a certain time *t*, *S* is the total effective area of membrane and *p* is the pressure. The porosity (*ε*) of the g-C_3_N_4_/TNA membranes was investigated by a gravimetric method and calculated using the following formula:

2 $$\varepsilon =\frac{{m}_{2}-{m}_{1}}{\rho V}\,$$

where *m*_1_ is the mass of dry membrane, *m*_2_ is the mass of wet membrane, *ρ* is the water density, *V* is the volume of membrane. The rejection rate was calculated by the formula:

3 $$R\,( \% )=(1-\frac{{C}_{p}}{{C}_{f}})\,\times 100\,$$

where *R*(%) is the percent solute rejection, and *C*_*p*_ and *C*_*f*_ are the concentrations of solute in the permeate and feed, respectively.

### Membrane performance tests

The performance of the g-C_3_N_4_/TNA membrane was investigated by the removal of RhB (3 mg · L^−1^). As shown in Fig. 4a, the test was performed in a paired-cell quartz reactor (with a volume of 30 mL). A piece of g-C_3_N_4_/TNA membrane was glued to a holder and mounted between the two half-cells. A 500 W xenon arc lamp (LSH-X500, Zolix, 100 mW · cm^−2^) was used as a light source with a 400 nm cut-off filter. The RhB solution was permeated the membrane under a pump (50 r/min). The concentration of RhB was determined by measuring the optical absorption at 554 nm with a JASCO UV-vis spectrophotometer (V550, Japan).

To evaluate the anti-biofouling capability of the g-C_3_N_4_/TNA membrane, the removal of *E. coli* was also investigated. Briefly, *E. coli* was incubated in Luria Bertani nutrient solution at 37 °C for 12 h with shaking and then washed with 0.9% saline. After centrifugation, the cell was re-suspended with 0.9% saline. The experiment of photocatalytic disinfection was carried out by using the set-up shown in Fig. 4a under the 500 W xenon arc lamp. NaCl was added into the feed water that contained *E. coli* and its concentration was adjusted at 0.9%. At the certain time intervals, 1 mL of suspension was sampled and immediately diluted 10-fold serially with sterilized saline. In order to measure the density of viable *E. coli* cell, 0.2 mL of the diluted solution was spread on the nutrient agar and incubated at 37 °C for 12 h. The number of cfu was counted to determine the number of viable cells. Each set of experiment was performed in triplicate and their average values with statistical deviation were used in the data analysis.

## Electronic supplementary material

Supporting information
