# Supplementary material for: Constructing a visible-light-driven photocatalytic membrane by g-C3N4 quantum dots and TiO2 nanotube array for enhanced water treatment
Source: Sci Rep. 2017 Jun 9;7:3128. doi: 10.1038/s41598-017-03347-y (PMC5466622; doi:10.1038/s41598-017-03347-y)
Supplement: Supplementary file 1 — Supporting information [file 41598_2017_3347_MOESM1_ESM.pdf]

Supporting information

Constructing a visible-light-driven photocatalytic membrane by g-C_3N_4 quantum dots and TiO_2 nanotube array for enhanced water treatment

Qi Zhang[1, 2], Xie Quan[2], Hua Wang*[1, 3], Shuo Chen*[2], Yan Su[2], Zhangliang Li[3]

[1]School of Fisheries and Life Science, Dalian Ocean University, Dalian 116023, China. [2]Faculty of Chemical, Environmental and Biological Science and Technology, Dalian University of Technology, Dalian 116024, China. [3]Fujian Provincial Key Laboratory of Ecology-Toxicological Effects & Control for Emerging Contaminants, Putian 351100, China.

Corresponding author. Hua Wang and Shuo Chen.

*E-mail address: wanghua@dlou.edu.cn (H. Wang); shuochen@dlut.edu.cn (S. Chen)

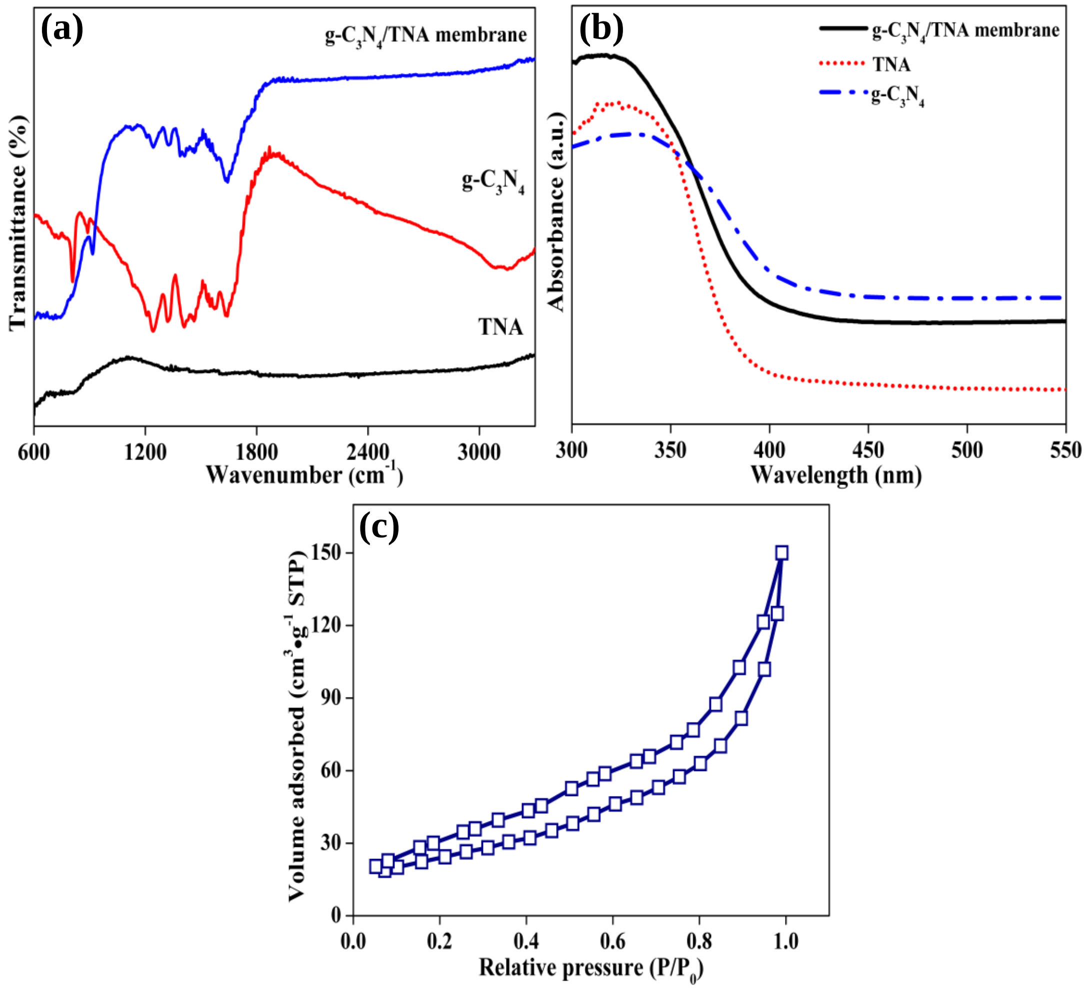

Figure S1. (a) FT-IR spectra and (b) UV-vis diffuse reflectance spectra of g-C_3N_4, TNA and the g-C_3N_4/TNA membrane; (c) N_2 adsorption/desorption isotherms of g-C_3N_4/TNA membrane 3.

As shown in Figure S1a, the g-C_3N_4 sample exhibited several strong characteristic peaks in the range of 1200-1700 cm^{-1} due to the typical stretching vibrations of C-N heterocycles[1]. The peak at 810 cm^{-1} corresponded to the characteristic breathing mode of the triazine units[2]. Because of the stretching vibration modes of terminal NH groups, a broad band in the range of 3150-3300 cm^{-1} was observed[3]. Meanwhile, the TNA exhibited a broad, characteristic absorption band from 500-700 cm^{-1}[4]. The main characteristic peaks corresponding to both g-C_3N_4 and TNA appeared in the g-C_3N_4/TNA membrane. UV-vis diffuse reflectance spectra are given in Figure S1b. Since the band gap of anatase TiO_2 is about 3.2 eV, the

absorption edge of TiO_2 is located at about 388 nm, which can only be excited by UV irradiation. The pristine g-C_3N_4 has an absorption edge of ca. 460 nm, corresponding to the optical bandgap of 2.70 eV. For the g-C_3N_4/TNA membrane, it can be seen from Figure S1b that the absorption edge extended to 418 nm upon introduction of g-C_3N_4 QDs. This result indicates that the obtained g-C_3N_4/TNA membranes can be stimulated by visible light. Figure S1c presents the calculated specific surface area of g-C_3N_4/TNA membrane is about 153 $m^2 \cdot g^{-1}$.

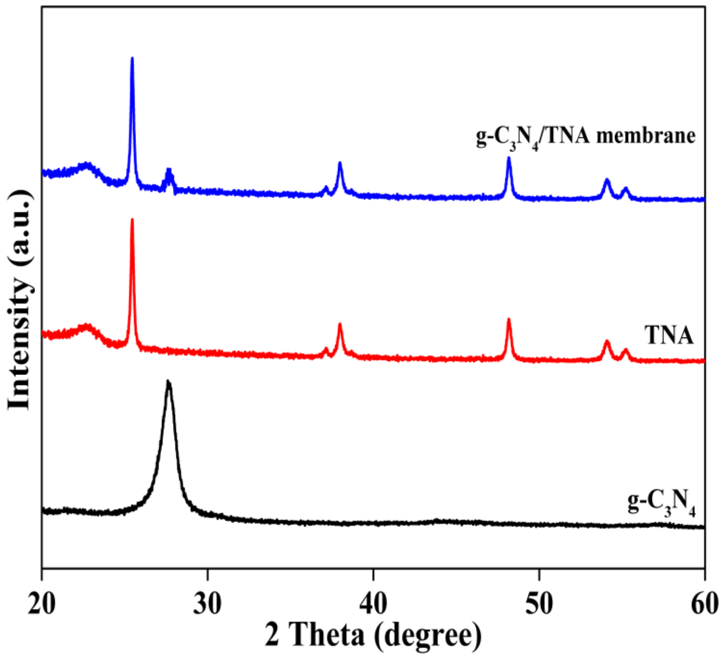

Figure S2. XRD patterns of sole g-C_3N_4, TNA and the g-C_3N_4/TNA membrane.

Figure S2 shows the XRD patterns of the g-C_3N_4/TNA membrane. Obviously, the same characteristic peaks of g-C_3N_4 and TiO_2 are observed at g-C_3N_4/TNA membrane compared with the sole g-C_3N_4 and TNA. It is evident that the g-C_3N_4 QDs were assembled into the TNA.

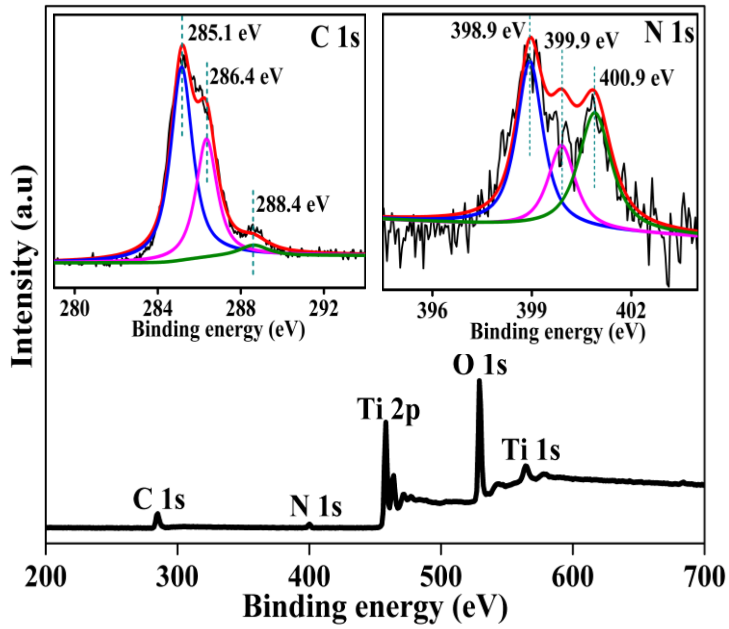

Figure S3. XPS spectra of the g-C_3N_4/TNA membrane (Inset: high-resolution spectra of C1s and N1s).

To analyze the chemical composition, the XPS spectra of the g-C_3N_4/TNA membrane were carried out. Figure S3 presents the chemical nature of the elements. The peaks of Ti, O, C and N were observed in the g-C_3N_4/TNA membrane. A high resolution C 1s spectrum was presented in inset of Figure S3 for the g-C_3N_4/TNA membrane and three peaks were found in order. The peak located at 285.1 eVcould be attributed to C–C and/or adventitious carbon. The peaks at 286.4 and 288.4 eV corresponded to the C–N–C and C–$(N)_3$ groups of g-C_3N_4, respectively[5]. Meanwhile, no peak corresponding to a chemical interaction between Ti and C (Ti–C) was seen for the g-C_3N_4/TNA membrane in the XPS spectrum (Figure S3). Thus, XPS revealed that there was no chemical interaction between the g-C_3N_4 and TNA. Inset of Figure S3 also shows the regional spectrum of N 1s for the g-C_3N_4/TNA membrane. The spectrum could be deconvoluted into three peaks *viz.* 398.9, 399.9, and 400.9 eV, which could account for C–N=C, N–$(C)_3$ and the –NH_2 or =NH of

non-condensed terminal amino groups within the material, respectively[6].

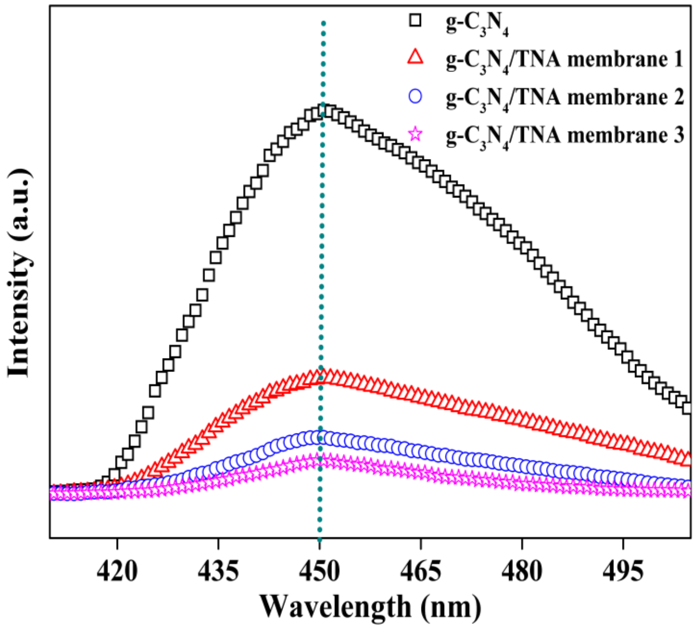

Figure S4. Photoluminescence spectra of $g\text{-}C_3N_4$ and $g\text{-}C_3N_4$/TNA membranes.

Figure S4 displays the photoluminescence spectra of $g\text{-}C_3N_4$ and $g\text{-}C_3N_4$/TNA membranes, and an evident emission peak is observed at about 450 nm, which is attributed to n-π* electronic transitions[7]. For the pure $g\text{-}C_3N_4$, its photoluminescence spectrum shows a strong emission from 410 to 500 nm. Meanwhile, the quenching of the intensity observed for all $g\text{-}C_3N_4$/TNA membranes indicated that the recombination of photogenerated electrons and holes could be suppressed. Moreover, the intensity of emission peak decreases from $g\text{-}C_3N_4$/TNA membrane 1 to 3, which means that the $g\text{-}C_3N_4$/TNA membrane 3 has a more favorable structure for the photocatalysis. Herein, benefiting from the heterojunction between the TNA membrane and the $g\text{-}C_3N_4$ QDs, the space charge region of the heterojunction would provide additional function for the photogenerated charge carriers' separation reducing the recombination of photogenerated electrons and holes due to the internal electrostatic field in the junction region, hence more

electrons and holes for photocatalytic reactions. Moreover, the large contacted area of TNA membrane with g-C_3N_4 QDs further accelerates the electron transfer rate. Hence, both the enhanced light absorption and lowered radiative electron-hole recombination endow this kind of g-C_3N_4/TNA membrane with high photocatalytic activity.

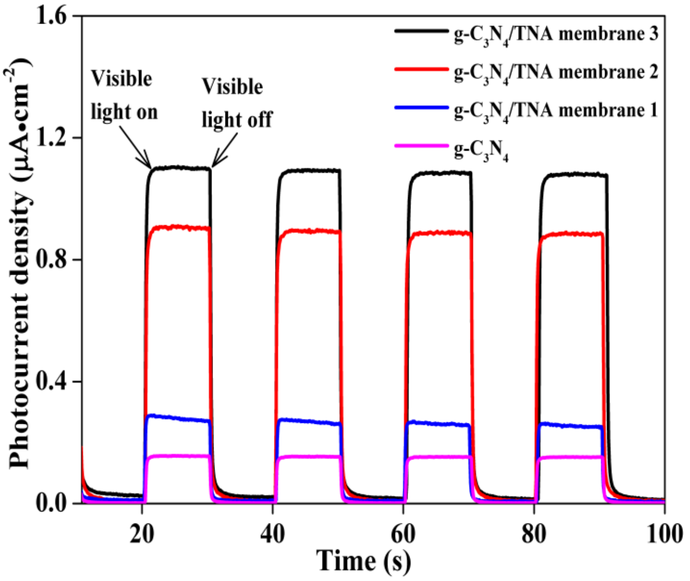

Figure S5. Photocurrent density of g-C_3N_4 and g-C_3N_4/TNA membranes.

The photocurrent density of sole g-C_3N_4, g-C_3N_4/TNA membranes 1, 2 and 3 is presented in Figure S5, which exhibit the separation and migration efficiency of the photogenerated carriers. Obviously, the current densityis negligible in dark. However, when the visible light switched on, a value of photocurrent density appeared. As shown in Figure S5, all four samples show a quick response to the light, but the photocurrent density is different. All the g-C_3N_4/TNA membranes present a higher photocurrent density than that of pure g-C_3N_4. Compared with g-C_3N_4/TNA membrane 1 (0.3 $\mu A \cdot cm^{-2}$) and 2 (0.9 $\mu A \cdot cm^{-2}$), the value of the photocurrent density of g-C_3N_4/TNA membrane 3 reached at 1.1 $\mu A \cdot cm^{-2}$ under the present experimental

conditions. This demonstrates that g-C_3N_4/TNA membrane 3 is superior to g-C_3N_4/TNA membrane 1 or 2 in photogenerated carriers' separation.

References

1. Yan, S. C., Li, Z. S. & Zou, Z. G. Photodegradation performance of g-C_3N_4 fabricated by directly heating melamine. *Langmuir* **25**, 10397–10401 (2009).

2. Zhang, Y., Thomas, A., Antonietti, M. & Wang, X. Activation of carbon nitride solids by protonation: Morphology changes, enhanced ionic conductivity, and photoconduction experiments. *J. Am. Chem. Soc.* **131**, 50–51 (2009).

3. Wang, X. *et al.* Metal-containing carbon nitride compounds: A new functional organic-metal hybrid material. *Adv. Mater.* **21**, 1609–1612 (2009).

4. Wang, W. K. *et al.* Photocatalytic degradation of atrazine by boron-doped TiO_2 with a tunable rutile/anatase ratio. *Appl. Catal. B-Environ.* **195**, 69–76 (2016).

5. Yan, S. C., Li, Z. S. & Zou, Z. G. Photodegradation of rhodamine B and methyl orange over boron-doped g-C_3N_4 under visible light irradiation. *Langmuir* **26**, 3894–3901 (2010).

6. Chang, F. *et al.* Fabrication, characterization, and photocatalytic performance of exfoliated g-C_3N_4-TiO_2 hybrids. *Appl. Surf. Sci.* **311**, 574–581 (2014).

7. Jorge, A. B. *et al.* H_2 and O_2 evolution from water half-splitting reactions by graphitic carbon nitride materials. *J. Phys. Chem. C* **117**, 7178–7185 (2013).
